# *BMP7* Functions to Regulate Proliferation of Dermal Papilla Cells in Hu Sheep

**DOI:** 10.3390/genes13020201

**Published:** 2022-01-22

**Authors:** Yue Li, Xiaoyang Lv, Shanhe Wang, Xiukai Cao, Zehu Yuan, Tesfaye Getachew, Joram M. Mwacharo, Aynalem Haile, Wei Sun

**Affiliations:** 1 College of Animal Science and Technology, Yangzhou University, Yangzhou 225009, China; yzudkly2019@163.com (Y.L.); shanhe12315@163.com (S.W.); 2 Joint International Research Laboratory of Agriculture and Agri-Product Safety of Ministry of Education of China, Yangzhou University, Yangzhou 225009, China; dx120170085@yzu.edu.cn (X.L.); cxkai0909@163.com (X.C.); yuanzehu1988@163.com (Z.Y.); 3 International Centre for Agricultural Research in the Dry Areas, Addis Ababa 999047, Ethiopia; T.Getachew@cgiar.org (T.G.); j.mwacharo@cgiar.org (J.M.M.); a.haile@cgiar.org (A.H.)

**Keywords:** *BMP7*, Hu sheep, dermal papilla cells, cell proliferation

## Abstract

Bone morphogenetic proteins (BMPs) are the structurally similar and highly conserved type of functional proteins that play an important role in hair follicle growth and development. *BMP7* was a differentially expressed gene in different patterns of Hu sheep lambskin identified using Agilent microarray. Since hair follicle is the basis of pattern formation of lambskin, and its growth and development is governed by dermal papilla cells (DPCs), to clarify the role of *BMP7* and hair follicle, our study was designed to investigate the regulation between *BMP7* and DPCs. Firstly, the CDS region of *BMP7* was cloned by 3’Race and PCR in Hu sheep and performed serious of bioinformatic analysis. Then, the effects of *BMP7* on DPCs were analyzed after overexpression and interference of *BMP7* in dermal papilla cells by CCK8, EdU, and PI assay. Additionally, qPCR was also conducted to clarify the relationship between *BMP7* and the TGF-β/Smad signaling pathway. A total of 1296 bp of the *BMP7* CDS region sequence was sucessfully cloned in Hu sheep, encoding a signal peptide of 431 amino acids, molecular weight was 49,316.9 Da and the isoelectric point (Pi) was 7.75. Nucleotide sequencing analysis of *BMP7* revealed that Hu sheep had high homology with *Bos taurus, Homo sapiens,* and *Canis lupus familiaris*. Structure domain prediction showed that TGF-β superfamily domain exist between 330th–431th amino acid, *BMP7* protein is a secreted protein. In *BMP7* up-regulated DPCs, DPCs proliferation rate and cell cycle were significantly higher than that of NC group (*p* < 0.05). Meanwhile, the expression level of *Smad3*, *Smad4*, *Samd6*, and *TGF-β1* in TGF-β/Smad signaling pathway were significantly lower than that in NC group (*p* < 0.05). In *BMP7* down-regulated DPCs, it presented the opposite result. In conclusion, our study showed that *BMP7* had a positive effect on DPCs by accelerating the proliferation and cell cycle of DPCs, and hypothesized that regulate hair follicles growth and development via TGF-β/Smad signaling pathway. These findings may provide a synergistic target for the subsequent research of hair follicle growth and development.

## 1. Introduction

Hu sheep, as a native Chinese sheep breed, is globally well known for its rare white lambskin. Pattern type, including large waves, medium waves, small waves and straight wool [1], is the leading indicator of lambskin quality. Mechanically, the density, fineness and curvature of wool are the key factors affecting the pattern type, which are largely determined by hair follicles. Early research has proven previous observations suggesting that hair follicles undergo complex processes of bidirectional epithelial-mesenchymal interactions in both embryonic and birth stages, the growth of hair follicle is governed by dermal papilla cells (DPCs), which can regulate the hair follicle cycle by promoting the proliferation of epithelial cells and inducing the differentiation of epithelial stem cells [2,3]. As was previously shown in mice, fur consists of different hair types, which are characterized by the number and type of DPCs in hair follicles [2,4,5]. Besides, several signaling pathways have been confirmed to be closely related to the growth and development of hair follicles, including Wnt/β-catenin [6] and TGF-β/BMP [7,8,9,10].

Bone morphogenetic proteins (BMPs), are a class of functional proteins with similar structure and high conservation. As a member of the TGF-β superfamily, it functions widely in diverse biological processes such as bone morphogenetic [11], nervous system [12], and hair follicles [13,14]. It is generally believed that BMPs have a suppressor function on the growth of hair follicles [15,16], meanwhile, antithetical effects have also been revealed. Previous report described BMPs can promote the growth cycle of hair follicles by acting as an inhibitor of Wnt/β-catenin signaling pathway [17]. Thus, BMPs may act both as a promoter or a suppressor in hair follicle growth. During the past decade, increasing members of BMPs have been identified as candidate genes in hair follicle growth and development, such as *BMP2* [18,19], *BMP4* [15], *BMP6* [7], and *BMP7* [20,21]. *BMP7* has been extensively studied for its function in growth and development of reproductive system [22], pituitary [23], ovary [24], and uterus [25]. Moreover, Noramly et al. reported that *BMP7* was associated with the size and spatial distribution of the feather germs [17], implying a potential role in hair follicle growth and development. In our previous research, transcriptomic profiles of the hair follicle of lambskin with large waves and small waves were obtained, and *BMP7* was revealed to act as a significantly different expressed gene [26]. Considering the extensive role of *BMP7* in diverse biological processes, we hypothesize that *BMP7* may also affect the cellular function of Hu sheep DPCs.

To evaluate this hypothesis, the *BMP7* CDS region sequence of Hu sheep was cloned and performed serious of bioinformatic analysis. Then further in-depth experiments were conducted to investigate the role of *BMP7* by examining expression profiles, effects on cell proliferation, cell cycle via overexpression and interference of *BMP7* in dermal papilla cells so that we could speculate the potential function of *BMP7* as a research candidate in hair follicle growth and development. Our study will provide some scientific basis for subsequent studies on the formation of wool curvature, thus laying the foundation for exploring the genetic mechanism of different pattern formation in lambskins.

## 2. Materials and Methods

### 2.1. Sample Collection and Cell Culture

Experimental lambs were supplied by Suzhou Stud Farm (Suzhou, Jiangsu Province, China). Around 1 cm^2^ of skin from the dorsal side of the 2-day-old Hu sheep lambs was collected and snap frozen in liquid nitrogen and then stored at −80 °C. Total RNA was extracted using Trizol (TIANGEN, Beijing, China) per the manufacturer’s instructions. First strand of cDNA was prepared using a PrimeScript^TM^ RT Reagent Kit according to the manufacturer’s instructions (Takara Bio, Beijing, China), and cDNA were stored at −20 °C.

Isolation, culture and identification of Hu lamb dermal papilla cells (DPCs) were followed with our laboratory methods [27]. DPCs were cultured in DMEM/F12 (Gibco, Grand Island, NY, USA) supplemented 10% fetal bovine serum (Sigma, St. Louis, MO, USA) with 5% CO_2_ at 37 °C.

All animal experimental procedures mentioned were approved by the Animal Care and Use Committee at Yangzhou University (NSFC2020-NFY-1).

### 2.2. Bioinformatics Analysis of BMP7 Gene in Hu Sheep

According to the sequences of *Bos Taurus* (NM_001206015.1), *Homo sapiens* (NM_001719.2) and *Mus musculus* (NM_007557.3), primers (1F, 1R) were designed to clone sheep *BMP7* partial CDS. On the basis of this, 3′RACE GSP inner primer (3I), 3′RACE GSP outer primer (3O) were designed to clone 3′UTR of *BMP7* after the partial CDS has been obtained (Table 1). Hu sheep *BMP7* full-length cDNA sequence was spliced by fragment overlapping areas, and then submitted to GenBank (No: KF925831).

Phylogenetic tree was computed using MEGA 11 software according to the neighbor-joining method based on the amino acid sequence of Hu sheep and other species such as *Homo sapiens*, *Bos Taurus*, *Mus musculus*, *Rattus norvegicus* (NM_001191856.1), *Danio rerio* (AF201379.1), *Tegillarca granosa* (JX103495.1), and *Canis lupus familiaris* (NM_001197052.1).

Basic chemical properties of proteins were analyzed using the ProtParam tool (ProtParam, http://web.expasy.org/protparam/, accessed on 22 December 2021); protein subcellular localization was analyzed using TargetP (TargetP-2.0, https://services.healthtech.dtu.dk/service.php?TargetP-2.0, accessed on 22 December 2021) and PSORT II Prediction (PSORT Prediction, http://psort.hgc.jp/form.html, accessed on 22 December 2021); potential signal peptide cleavage sites prediction were performed using SignalP4.1 (SignalP4.1, https://services.healthtech.dtu.dk/service.php?SignalP-4.1, accessed on 22 December 2021); glycosylation sites were analyzed using NetOGlyc4.0 (NetOGlyc4.0, https://services.healthtech.dtu.dk/service.php?NetOGlyc-4.0, accessed on 22 December 2021); the phosphorylation sites of amino acids were predicted using NetPhos3.1 (NetPhos-3.1, https://services.healthtech.dtu.dk/service.php?NetPhos-3.1, accessed on 22 December 2021); conserved domains of the amino acid sequence were predicted using Smart (SMART, http://smart.embl-heidelberg.de/, accessed on 22 December 2021); the hydrophilicity of amino acid sequences was analyzed using ProtScale (ProtScale, http://web.expasy.org/protscale/, accessed on 22 December 2021); protein secondary structure was predicted using GOR IV (GOR IV SECONDARY STRUCTURE PREDICTION METHOD, http://npsa-pbil.ibcp.fr/cgi-bin/npsa_automat.pl?page=npsa_gor4.html, accessed on 22 December 2021); transmembrane analysis of protein sequences was performed using TMHMM-2.0 (TMHMM-2.0, https://services.healthtech.dtu.dk/service.php?TMHMM-2.0, accessed on 22 December 2021). 

### 2.3. Over Expression and Inhibition of BMP7

The CDS sequence of *BMP7* gene was amplified from the cDNA by PCR using the gene-specific primers designed from the Hu sheep *BMP7* gene. The restriction sites of Xho Ⅰ and Kpn Ⅰ (underlined in the sequences below) were introduced into the forward primer (5′-ccCTCGAGATGCACATGCGCTCGCTAC-3′) and the reverse primer (5′- ggGGTACCCTAGTGGCAGCCACAAGCCC -3′), respectively. The PCR product was gel-purified, sequenced and ligated into pEX-1 vector using DNA Ligation Kit (Takara Bio, Beijing, China).

Small interfering RNA against *BMP7* (siRNA-*BMP7*) were designed and synthesized by GenePharma (Shanghai, China), details were provided in Table 2.

pEX-1-*BMP7* and siRNA-*BMP7* were transfected into DPCs respectively using jetPRIME transfection reagent (Polyplus-transfection, Illkirch-Graffenstaden, France). DPCs were collected for following studies at 24 h after transfections, including Negative control (NC).

### 2.4. Cell Proliferation and Cycle Assay

The DPCs were transferred to 96-well plates before cell proliferation assay, 10 μL Cell Counting Kit-8 (CCK-8) solution was added to each well at 0, 24, 48 and 72 h after transfections. The OD value was measured at 450 nm with a Tecan Infinite F200/M200 microplate reader (Tecan, *Männedorf*, Switzerland) after 2 h. The proliferation of DPCs was also detected at 24 h after transfections using the Cell-Light EdU Apollo567 In Vitro Kit (RiboBio, Guangzhou, China) according to the manufacturer’s instructions.

The cell cycle of DPCs was also detected at 24 h after transfections using Cell Cycle and Apoptosis Analysis Kit (Beyotime, Shanghai, China) according to the manufacturer’s instructions by flow cytometry.

### 2.5. qRT-PCR

After overexpression and disruption of *BMP7* in dermal papilla cells, several key genes (*Smad1, Smad3, Smad4, Smad5, Smad6,* and *TGF-β1*) in the TGF-*β*/Smad signaling pathway were selected for qRT-PCR.Total RNA was extracted and first strand of cDNA was prepared. Based on assembled sequences of studied genes in GenBank, primers were designed using the Primer Premier v5.0 software (Table 3). All primers were synthesized by Sango Biotechnology Co., Ltd. (Beijing, China). Taking Lu et al. s’ research as a reference [28], house-keeping gene (*GAPDH*) was used as an internal control to normalize the threshold cycle (CT) values. The 2^−ΔΔCT^ method was used to process real-time PCR results [29].

### 2.6. Western Bolt

After the DPCs were transfected for 36 h, the protein of DPCs were disposed using RIPA Lysis Buffer (Beyotime, Shanghai, China), protein concentrations were measured using Enhanced BCA Protein Assay Kit (Beyotime, Shanghai, China). The proteins were separated by SDS-PAGE, transferred to polyvinylidene difluoride (PVDF) membranes, and probed with 1:500 rabbit anti-*BMP7* (Huabio, Hangzhou, China) and 1:2500 rabbit anti-*GAPDH* (Proteintech Group, Rosemont, IL, USA) antibodies, and then with 1:3000 goat anti-rabbit IgG HRG antibodies (ABclonal, Wuhan, China). Signals were detected with the enhanced chemiluminescence ECL Western Blot kit (Biosharp, Hefei, China). All experimental producers were performed per the manufacturer’s instructions. Protein was detected and analyzed by the ChemiDoc^TM^ Analysis System (Bio-Rad, Hercules, CA, USA).

### 2.7. Statistical Analysis

All mentioned experiments were performed in triplicate to ensure the reliability of our study. The 2^−ΔΔCT^ method was used to process real-time PCR results [29]. Statistical analyses were carried out using SPSS 18.0 software (IBM, Armonk, NY, USA). Independent sample t-test and one-way analysis of variance (ANOVA) were used to perform variance analysis and significance test. All experimental data are presented as mean ± standard error of the mean (SEM). A probability of *p* ≤ 0.05 was considered statistically significant, and a probability of *p* ≤ 0.01 was considered to be extremely statistically significant. 

## 3. Results

### 3.1. Cloning Hu Sheep BMP7 Full Length cDNA Sequence

The 478 bp CDS sequence was successfully cloned by 1F and 1R amplification on cDNA. 3’ RACE was used to clone 1095 bp sequence. A total of 1296 bp CDS region sequence was obtained after spliced these two sequences according to overlapping sequence, and submitted to GenBank (No: KF925831).

### 3.2. Bioinformatics Analysis of BMP7 Gene in Hu Sheep

**Figure 1:**
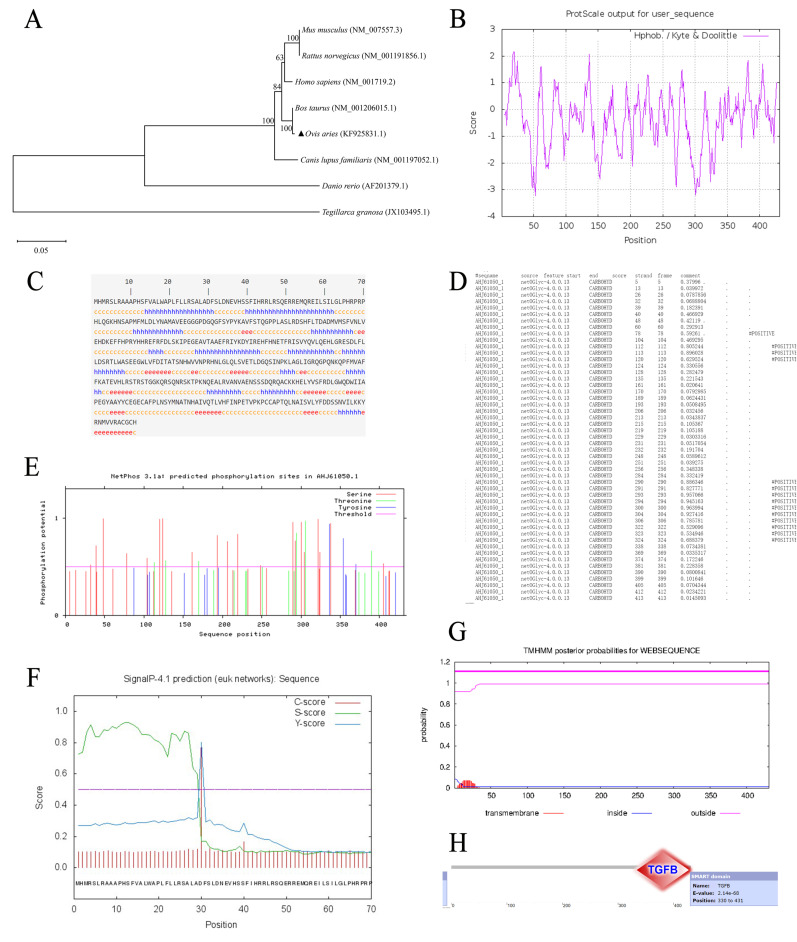
Bioinformatics analysis of *BMP7* gene in Hu sheep. (**A**) Analysis of phylogenetic tree for *BMP7* gene. (**B**) The hydropathy profile of *BMP7* sheep amino acid. Note: The horizontal scale indicated the number of amino acid residues and the vertical one was the relative hydropathy scale. Points above the zero horizontal line corresponded to hydrophobic region, and points below the line were hydrophilic. (**C**) *BMP7* protein secondary structure prediction. (**D**) *BMP7* glycosyl site analyses. (**E**) *BMP7* phosphorylation site analyses. (**F**) *BMP7* signal peptide prediction. (**G**) *BMP7* protein transmembrane regional analyses. (**H**) *BMP7* conservative structure domain analyses.

Similarity analysis was computed on nucleotide and amino acid sequence of Hu sheep’s *BMP7* CDS region and *Homo sapiens, Bos Taurus, Mus musculus, Rattus norvegicus, Danio rerio, Tegillarca granosa,* and *Canis lupus familiaris* (Table 4). The results of nucleotide similarity analysis showed the highest homology existed between Hu sheep and *Bos Taurus* (98.5%), followed by *Homo sapiens* (92.7%), the results of amino acid similarity analysis showed the highest homology existed between Hu sheep and *Bos Taurus* (99.4%), followed by *Mus musculus,* and *Rattus norvegicus* (98.9%)*,* phylogenetic tree was also constructed based on amino acid sequence (Figure 1A), which also suggested a high similarity between these species.

As is mentioned above, serious of bioinformatics analysis were performed. The CDS region of Hu sheep’s *BMP7* was 1296 bp, encoding a signal peptide of 431 amino acids, molecular weight was 49,316.9Da and the isoelectric point (Pi) was 7.75. Most of the amino acid sequence contained hydrophilic residues, which indicated that Hu sheep’s *BMP7* protein was water-soluble (Figure 1B). Result of subcellular localization showed that *BMP7* protein was a secreted protein (Table 5). Secondary structure prediction of components of *BMP7* protein showed that α-helix (h) accounted for 34.11%, β-sheet (e) accounted for 14.62%, and random coils (c) accounted for 51.28% (Figure 1C). Structure prediction revealed that *BMP7* protein had 14 potential glycosylation sites (Figure 1D), 30 potential phosphorylation sites (Figure 1E), a signal peptide cleavage site between 29th–30th (Figure 1F), and no transmembrane helices structure in *BMP7* protein (Figure 1G). Further in-depth structure domain prediction showed that TGF-β superfamily domain existed between 330th–431th amino acid (Figure 1H). Functional analysis of *BMP7* protein revealed that *BMP7* protein was major enriched in biosynthesis of cofactors, transport and binding, and translation, details were shown in Table 6.

### 3.3. BMP7 Promoted Proliferation of Hu Sheep DPCs

**Figure 2:**
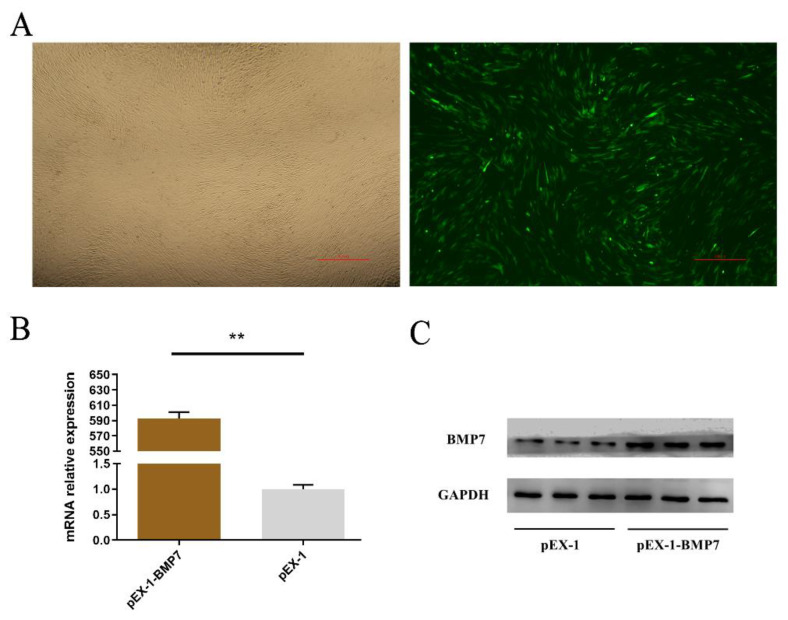
Validation of *BMP7* transfection efficiency. (**A**) Transfection efficiency by green fluorescent protein. (**B**) The mRNA expression level of *BMP7*. Note: “**” represents an extremely significant difference (** *p* < 0.01). (**C**) The protein level of *BMP7*.

**Figure 3:**
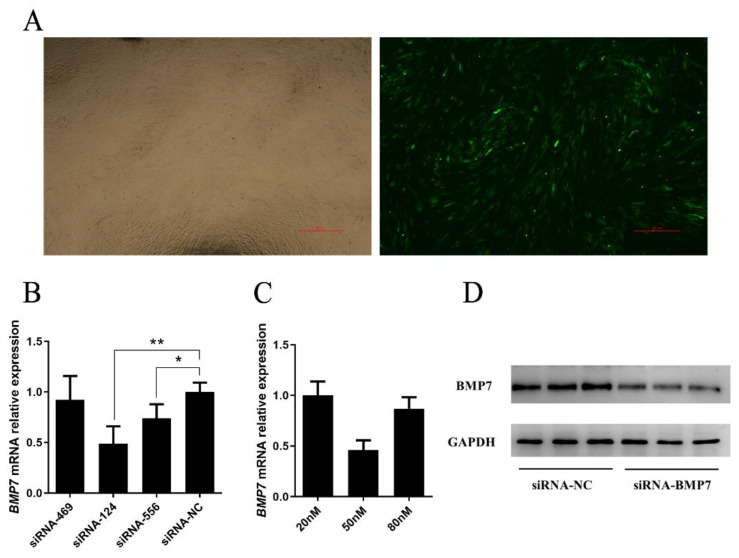
The mRNA and protein level of *BMP7* in dermal papilla cells transfected with siRNA-*BMP7*. (**A**) Transfection efficiency by green fluorescent protein. (**B**,**C**) The mRNA expression level of *BMP7*. (**D**) The protein level of *BMP7*. “*” represents a significant difference (*p* < 0.05) and “**” represents an extremely significant difference (*p* < 0.01).

To estimate the role of *BMP7* in DPCs, we designed over-expression vector (pEX-1-*BMP7*) and siRNA (siRNA-*BMP7*s) specifically targeted *BMP7*. First, we verified the transfection effects of pEX-1-*BMP7* and siRNA-*BMP7*s, at 24 h post transfection, both pEX-1-*BMP7*-transfected clones and siRNA-*BMP7*s transfected clones showed clearly visible fluorescence signal (Figure 2A and Figure 3A), the results of RT-qPCR (Figure 2B) and Western bolt (Figure 2C) showed that pEX-1-*BMP7* can up-regulate the expression of *BMP7* at mRNA and protein level. Regarding siRNAs, detectable interfering mRNA levels of *BMP7* were observed after the transfection of all three siRNA-*BMP7*s (Figure 3B), within which, the transfection effects of siRNA-124 were higher compared with other control-transfected siRNAs at statistically significant level (*p* < 0.05), and lowest level of *BMP7* mRNA expression were obtained with siRNA-124 at a concentration of 50 nM (Figure 3C). Furthermore, the results of Western bolt (Figure 3D) showed that the transfection of siRNA-124 at a concentration of 50 nM can down-regulate the expression of *BMP7* protein. Collectively, our results showed that *BMP7* could be successfully over/down expressed in DPCs by transfection.

**Figure 4:**
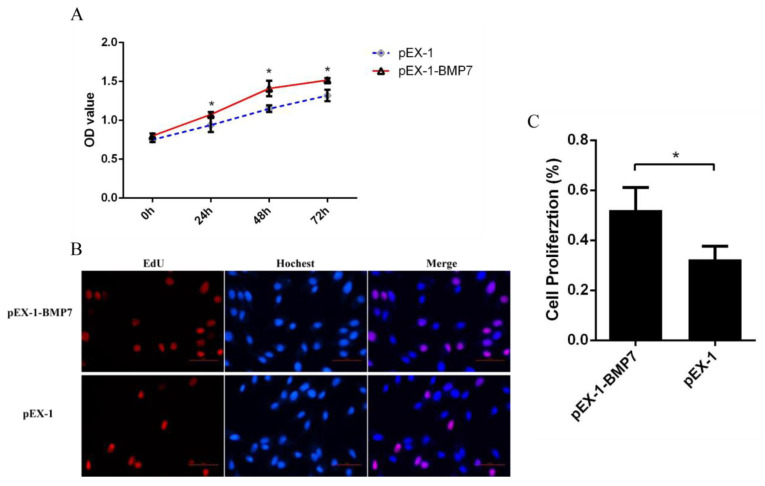
The effects of *BMP7* over-expression on dermal papilla cell proliferation of Hu sheep. (**A**) Detection of dermal papilla cell activity by CCK-8 after over-expression of *BMP7* gene. (**B**) Detection of dermal papilla cell proliferation by EdU after over-expression of *BMP7* gene. (**C**) The rate of proliferating cells. “*” represents a significant difference (*p* < 0.05).

**Figure 5:**
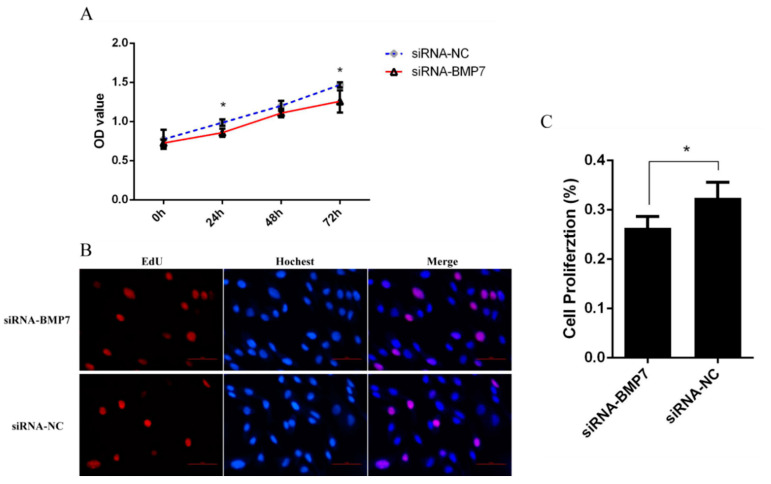
The effects of *BMP7* down-regulation on dermal papilla cell proliferation of Hu sheep. (**A**) Detection of dermal papilla cell activity by CCK-8 after *BMP7* down-regulation. (**B**) Detection of dermal papilla cell proliferation by EdU after *BMP7* down-regulation. (**C**) The rate of proliferating cell. “*” represents a significant difference (*p* < 0.05).

**Figure 6:**
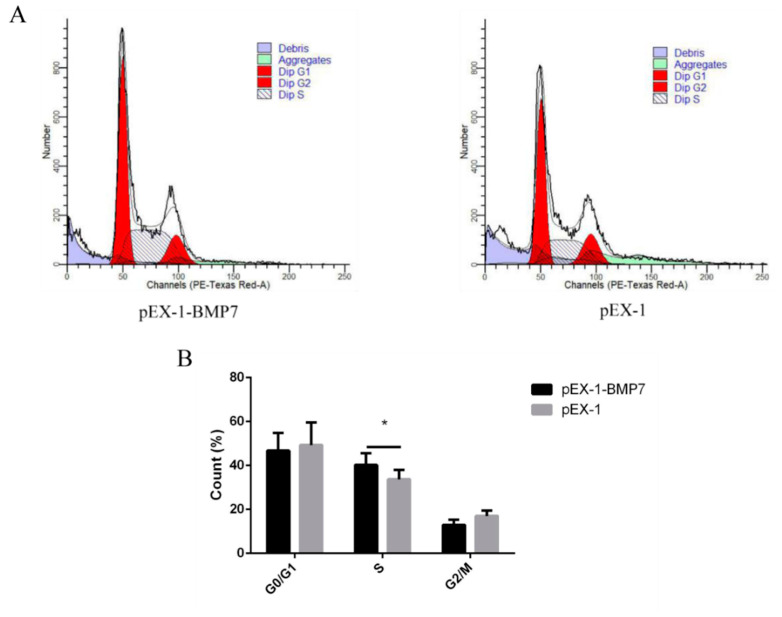
The effects of dermal papilla cell cycle after *BMP7* over-expression. (**A**) The dermal papilla cell cycle after *BMP7* over-expression. (**B**) The rate of different periods of the cell cycle. “*” represents a significant difference (*p* < 0.05).

**Figure 7:**
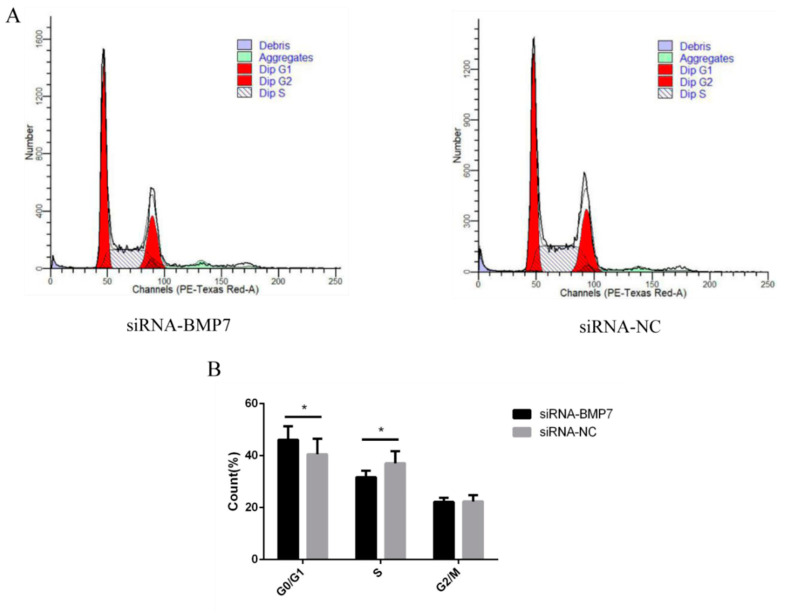
The effects of dermal papilla cell cycle after *BMP7* down-regulation. (**A**) The dermal papilla cell cycle after *BMP7* down-regulation. (**B**) The rate of different periods of the cell cycle. “*” represents a significant difference (*p* < 0.05).

To determine whether *BMP7* affects DPCs proliferation, we quantified cell proliferation and cycle of DPCs. As measured by CCK-8 assay, the proliferation rate was significantly higher in pEX-1-*BMP7*-transfected DPCs than that of negative control (pEX-1 transfected) DPCs (Figure 4A), as expected, lower proliferation rate was revealed in siRNA-124-transfected DPCs than that of non-targeting siRNA-transfected DPCs, especially at 24 and 72 h post transfection (Figure 5A). Results were similar in Edu assay, the transfection of pEX-1-*BMP7* can significantly promote the proliferation of DPCs (Figure 4B,C), and cell proliferation can be inhibited by the downregulation of *BMP7* in DPCs (Figure 5B,C). Next, the cell cycle was analyzed by flow cytometry. The results showed that proportion of cells in S phase were notably higher in pEX-1-*BMP7*-transfected DPCs compared to NC (Figure 6), and lower proportion of cells in S phase were observed in siRNA-124-transfected DPCs compared to NC (Figure 7).

### 3.4. BMP7 Regulated Key Genes in TGF-β/Smad Signaling Pathway

**Figure 8:**
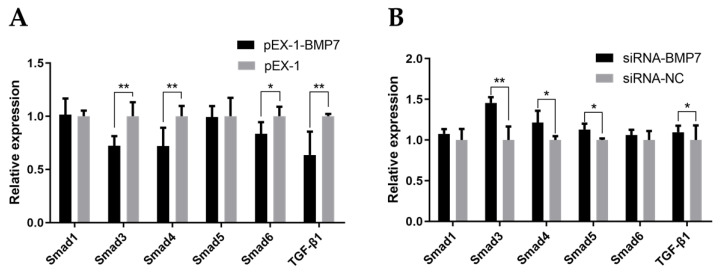
The gene expression of the TGF-β/Smad signaling pathway. (**A**) The effects of *BMP7* over-expression on the gene expression of the TGF-β/Smad signaling pathway. (**B**) The effects of *BMP7* interference on the gene expression of the TGF-β/Smad signaling pathway. “*” represents a significant difference (*p* < 0.05) and “**” represents an extremely significant difference (*p* < 0.01).

Given that TGF-β/Smad signaling pathway was involved in diverse cellular biological processes and the crucial role of *BMP7* in TGF-β/Smad signaling pathway, we evaluated the mRNA expression levels of the key genes in the TGF-β/Smad signaling pathway (*Smad1*, *Smad3*, *Smad4*, *Smad5*, *Smad6*, and *TGF-β1*) after transfection using qRT-PCR. As shown in Figure 8A, the expression level of *Smad3*, *Smad4*, *Smad6*, and *TGF-β1* were significant lower in pEX-1-*BMP7*-transfected DPCs compared to NC (*p* < 0.05), whereas the expression levels of *Smad1* and *Smad5* showed non-significant difference. After down-regulating *BMP7* in DPCs (Figure 8B), the expression levels of *Smad3*, *Smad4*, *Smad5*, and *TGF-β1* were significantly up regulated (*p* < 0.05), non-significant difference was noted in the expression levels of *Smad1* and *Smad6*. Besides, In *BMP7* up-regulated DPCs and *BMP7* down-regulated DPCs groups, notably, *Smad3*, *Smad4*, *Smad6*, and *TGF-β1* genes showed the opposite trends after transfection.

## 4. Discussion

The quality of lambskin in Hu sheep is influenced by the pattern type, which is closely associated with the wool curvature. Previous research has found that it can lead to pressure on one side of the wool fiber, ultimately giving rise to wool curvature, when the cell proliferation was asymmetric on both sides of hair follicle. To a certain degree, the greater the asymmetry of cell proliferation, the greater the pressure, and the more obvious the bending degree of wool fiber. Therefore, the asymmetry of cell proliferation in hair follicles was considered to be a significant factor affecting wool curvature [30]. Multiple growth factors and intercellular signaling molecules, expressed and secreted by dermal papilla cells, had an impact on the formation of hair follicle symmetry/asymmetry axis, including Wnt, TGF-β/BMP, Notch, Shh, FGFs and other regulatory factors. Kwack et al. reported that these factors regulated the proliferation and differentiation of cells in hair follicles by acting on neighboring cells through paracrine pathway [31]. In addition, Nissimov et al. proposed the hypothesis of “Multiple papillary centers” (MPC), which explained the cytological mechanism of hair bending [32]. MPC, referred to two or more dermal papilla regions, and the hypothesis shown that these dermal papillae can induce the growth rate of different cortical cells in their adjacent areas independently, leading to the difference of cell growth rate and eventually resulting in hair bending. In view of the effects of dermal papilla cells on the proliferation and differentiation of various cells in hair follicles, even on hair bending, we carried out experiments with dermal papilla cells as the core.

As is described in our preliminary transcriptomic data, *BMP7*, one of the most biologically active proteins in the BMP family, was identified as significant differently expressed gene between the large waves and small waves of the hair follicle in Hu sheep lambskin [26]. Existing studies have proved that BMP family can regulate the proliferation, apoptosis and differentiation of cells in hair follicles and play respective roles in different cells, finally affecting the growth and development of hair follicles [13,33]. It has been found that overexpression of *BMP2* increased the expression of differentiation marker factors and decreased the expression of proliferation marker factors in hair follicle stem cells, which promoted the differentiation process of hair follicle stem cells [18,19]. Wu et al. reported that *BMP6* regulated the transition of hair follicle from telogen phase to anagen phase through acting as an inhibitor of hair follicle stem cell activation [7], and the similar inhibitory effect was also found in *BMP4* on hair follicle growth and development [15,16,34]. It is worth mentioning that the expression levels of *BMP4* and *BMP6* would be elevated while hair follicles were activated and began to grow [35,36], which appeared to be contrary to the previously discovered theory that BMPs signal was an inhibitory signal [7,15,16,34]. Nevertheless, some researchers considered it was a negative feedback loop after in-depth understanding [35,36]. Although no evidence that *BMP7* can directly regulate wool curvature have been reported yet, several findings suggested that *BMP7* played a direct role in the growth and development of hair follicles [37,38]. A more general involvement of *BMP7* in the development of hair follicle and thereby epithelial–mesenchymal interactions was indicated by its strong expression in developing hair follicles, and the concomitant morphological deformation of structure [38]. Apparently, *BMP7* could participate in the formation of several traits in wool, but the specific regulatory mechanism is unclear. Considering the critical role of dermal papilla cells, and the participation of *BMP7* in hair follicle growth and development, we can hypothesis that *BMP7* may also play a role in the hair follicle growth and development by regulating the cellular process of DPCs, hence our study was designed and tried to provide an insight into the cellular function of *BMP7* in DPCs and further clarify the role of *BMP7* in hair follicle growth and development.

In our present study, in *BMP7*-upregulated DPCs, proliferation rate was promoted and the cell cycle was driven more rapidly. Meanwhile, proliferation rate and cell cycle was inhibited by down regulation of *BMP7*. In contrast to most previous findings, which had suggested suppressor function of BMPs in hair follicle growth and development [15,16], our study investigated that *BMP7* can promote cell proliferation and driven cell cycle of Hu sheep DPCs. In order to explain these differences, one should take into consideration that the negative feedback regulation and increased expression of *BMP4* in the early stage of hair follicle growth [35,36], hence we assume that negative feedback regulation may also exist between *BMP7* and hair follicle growth. By contrast, it is worth noting that our results are closer to Adly et al. s’ research [21]. As shown in their study on the relationship between *BMP7* protein and human hair follicle cycling, *BMP7* was found highly expressed during the growth phase of hair follicle, including outer root sheath, inner root sheath, fur layer cells, dermal papilla, and connective tissue sheath. Additionally, high expression level of *BMP7* was also detected in these cells during the proliferation phase of hair follicle cells. As hair follicle cells proliferate to the catagen and telogen phase during which *BMP7* was always found expressed, then *BMP7* was no longer expressed in most hair follicle cells, and the expression of *BMP7* showed a corresponding decrease in dermal papilla. Combing our data with predecessors’ reports, it is reasonable to believe that *BMP7* can participate in the growth and development of hair follicles of Hu sheep through promoting cell proliferation and driving cell cycle of DPCs, and may even affect the curvature of wool and the formation of lambskin pattern.

Known signaling pathways such as Wnt/β-catenin, EDA/EDAR, TGF-β/Smad and Notch interrelate and interact with each other to form a complex network to regulate the occurrence and cycle of hair follicles, and then affect hair traits [39,40]. The mechanisms associated with TGF-β/Smad signaling pathway related to BMPs have been partially explained in recent studies. It has been found that decorin (DCN) had a certain impact on the morphogenesis and periodic changes of hair follicles by way of promoting TGF-β and BMP signal transduction pathway [9]. Meanwhile, activation of *Smad4* in TGF-β/Smad signaling pathway can lead to hair fiber keratinization [41], and activate expression of downstream target genes, further affecting proliferation and differentiation of epithelial cells [42]. In addition, Song et al. reported that the low expression of *BMP2* and BMPR-IA in the telogen phase supported their inhibitory effect on the growth of hair follicle cells in yak [8]. *BMP7* was reported to act on TGF-β/Smad signaling pathway to realize signal transduction via binding to the BMPs receptors (BMPRs) on the cell membrane [43]. To evaluate the connection between *BMP7* and TGF-β/Smad signaling pathway in hair follicle growth, we performed the qPCR to measure the mRNA expression level of several key candidate genes in TGF-β/Smad signaling pathway (*Smad1*, *Smad3*, *Smad4*, *Smad5*, *Smad6*, *and TGF-β1*). In *BMP7* up/down-regulated DPCs, the results showed that *Smad3*, *Smad4*, *Smad6*, and *TGF-β1* genes showed the opposite pattern, however, non-significant difference was noted in the expression level of *Smad1*. In light of the aforementioned observations, we could speculate that *BMP7* may regulate hair follicle growth via influencing the signal transduction pathway or key candidate genes of TGF-β/Smad signaling pathway. Of course, further research is needed to confirm our speculation. In conclusion, our study shown that overexpression of *BMP7* resulted in accelerating the proliferation and cell cycle of DPCs, which can also be inhibited by downregulation of *BMP7*. Furthermore, qPCR results suggested that *BMP7* may regulate hair follicle growth via TGF-β/Smad signaling pathway. Given the important roles of BMPs, which interact with the TGF-β/Smad signaling pathway, our novel findings suggest a new direction for research of hair follicle growth and development, and can provide molecular basic for the breeding of Hu sheep.

## Figures and Tables

**Table 1:** The primer for cloning of *BMP7*.

Gene name	Primer Name	Sequences (5′→3′)	Length (bp)
*BMP7*	1F	TAGAGCCGGCGCGATGCAC	478
	1R	ACGCTACCACCACCGGGAG	
	3O	CCTATCCCTACAAGGCCGTCT	1466
	3I	ATCACAGCCACCAGCAACCAC	1095

**Table 2:** The sequence information of siRNA-*BMP7*.

Name	Sequences (5′→3′)
siRNA-124	sense: GCACUCGAGCUUCAUCCAUTT
	antisense: AUGGAUGAAGCUCGAGUGCTT
siRNA-469	sense: CCACCCGGGAGUUCCGGUUUTT
	antisense: AAACCGGAACUCCCGGUGGTT
siRNA-556	sense: CCGGGAACACUUCCACAAUTT
	antisense: AUUGUGGAAGUGUUCCCGGTT

**Table 3:** The primer information of qRT-PCR.

Gene Name	Sequences(5′→3′) (F: Forward R: Reverse)	Length (bp)	Tm (°C)	Accession No.
*BMP7*	F: TGAGTTCCGCATTTACAAGG	177	60	KF925831.1
	R: GTGGCTGTGATGTCAAAAAC			
*Smad1*	F: GAAAGCCCCGTTCTTCCTCC	150	60	AY035385.1
	R: GTTGGGCTGCTGGAAAGAAT			
*Smad3*	F: GGACGACTACAGCCATTCCA	172	60	AF508024.1
	R: ATTCGGGGAGAGGTTTGGAG			
*Smad4*	F: CTTCAGGTGGCTGGTCGG	177	60	NM_001267886.1
	R: TCCAGGTGATACAACTCGTTCA			
*Smad5*	F: CCAGTATATCCAGCAGAGATGTT	102	60	AF508027.1
	R: AAGCTTCCCCAACACGATTG			
*Smad6*	F: CTGCTCGGACGCCTCTTC	105	60	XM_004010255.5
	R: GGGTGGCGGTGATTCTGG			
*TGF-β1*	F: GGTGGAATACGGCAACAAAATC	162	60	NM_001009400.2
	R: TGCTGCTCCACTTTTAACTTGA			
*GAPDH*	F: GTCGGAGTGAACGGATTTGG	196	60	NM_001190390.1
	R: CATTGATGACGAGCTTCCCG			

Note: Bone morphogenetic protein 7 (*BMP7*), SMAD family member 1 (*Smad1*), SMAD family member 3 (*Smad3*), SMAD family member 4 (*Smad4*), SMAD family member 5 (*Smad5*), SMAD family member 6 (*Smad6*), transforming growth factor beta 1 (*TGF-β1*), glyceraldehyde-3-phosphate dehydrogenase (*GAPDH*).

**Table 4:** Similarity analysis of nucleotide and amino acid of *BMP7*.

Species	Accession No.	Nucleotide (%)	Amino Acid (%)
*Homo sapiens*	NM_001719.2	92.7%	98.7%
*Bos taurus*	NM_001206015.1	98.5%	99.4%
*Mus musculus*	NM_007557.3	89%	98.9%
*Rattus norvegicus*	NM_001191856.1	88%	98.9%
*Danio rerio*	AF201379.1	68%	88.4%
*Tegillarca granosa*	JX103495.1	48.7%	79.5%
*Canis lupus familiaris*	NM_001197052.1	92.1%	98.5%

**Table 5:** Subcellular localization of *BMP7*.

Name	Length	mTP	SP	Other
AHJ61050.1	431	0.327	0.858	0.015

**Table 6:** The function analysis of *BMP7* protein.

Functional Category	Odds
Amino acid biosynthesis	0.500
Biosynthesis of cofactors	2.917
Cell envelope	0.541
Cellular processes	0.411
Central intermediary metabolism	0.762
Energy metabolism	0.389
Purines and pyrimidines	1.308
Fatty acid metabolism	1.362
Replication and transcription	0.211
Regulatory functions	0.075
Translation	1.614
Transport and binding	1.885

## Data Availability

The raw data supporting the conclusions of this article will be made available by the authors, without undue reservation.
